# Importance of Sequencing *HBA1*, *HBA2* and *HBB* Genes to Confirm the Diagnosis of High Oxygen Affinity Hemoglobin

**DOI:** 10.3390/genes13010132

**Published:** 2022-01-12

**Authors:** Mathilde Filser, Betty Gardie, Mathieu Wemeau, Patricia Aguilar-Martinez, Muriel Giansily-Blaizot, François Girodon

**Affiliations:** 1 Biology Division, Department of Biological Hematology, Dijon Hospital, 21000 Dijon, France; mathildefilser@hotmail.fr; 2 Thorax Institute, Nantes Hospital, CNRS, Inserm, Nantes University, 44000 Nantes, France; betty.gardie@inserm.fr; 3 Ecole Pratique des Hautes Etudes, EPHE, Science and Letters Paris University, 75014 Paris, France; 4 Laboratory of Excellence GR-Ex, 75015 Paris, France; 5 Blood Disease Department, Claude Huriez Hospital, Lille Hospital, 59000 Lille, France; Mathieu.WEMEAU@CHRU-LILLE.FR; 6 Department of Biological Hematology, Saint Eloi Hospital, Montpellier Hospital & Montpellier University, 34000 Montpellier, France; p-martinez@chu-montpellier.fr (P.A.-M.); m-giansily@chu-montpellier.fr (M.G.-B.); 7 Inserm U1231, Bourgogne University, 21000 Dijon, France

**Keywords:** high oxygen affinity hemoglobin, erythrocytosis, next generation sequencing, P50, *HBB*, *HBA1*, *HBA2*

## Abstract

High oxygen affinity hemoglobin (HOAH) is the main cause of constitutional erythrocytosis. Mutations in the genes coding the alpha and beta globin chains (*HBA1*, *HBA2* and *HBB*) strengthen the binding of oxygen to hemoglobin (Hb), bringing about tissue hypoxia and a secondary erythrocytosis. The diagnosis of HOAH is based upon the identification of a mutation in *HBA1*, *HBA2* or *HBB* in specialized laboratories. Phenotypic studies of Hb are also useful, but electrophoretic analysis can be normal in 1/3 of cases. The establishment of the dissociation curve of Hb can be used as another screening test, a shift to the left indicating an increased affinity for Hb. The direct measurement of venous P50 using a Hemox Analyzer is of great importance, but due to specific analytic conditions, it is only available in a few specialized laboratories. Alternatively, an estimated measurement of the P50 can be obtained in most of the blood gas analyzers on venous blood. The aim of our study was therefore to determine whether a normal venous P50 value could rule out HOAH. We sequenced the *HBB*, *HBA1* and *HBA2* genes of 75 patients with idiopathic erythrocytosis. Patients had previously undergone an exhaustive medical check-up after which the venous P50 value was defined as normal. Surprisingly, sequencing detected HOAH in three patients (Hb Olympia in two patients, and Hb St Nazaire in another). A careful retrospective examination of their medical files revealed that (i) one of the P50 samples was arterial; (ii) there was some air in another sample; and (iii) the P50 measurement was not actually done in one of the patients. Our study shows that in real life conditions, due to pre-analytical contingencies, a venous P50 value that is classified as being normal may not be sufficient to rule out a diagnosis of HOAH. Therefore, we recommend the systematic sequencing of the *HBB*, *HBA1* and *HBA2* genes in the exploration of idiopathic erythrocytosis.

## 1. Introduction

Erythrocytosis and polycythemia are generic terms referring to red cell diseases characterized by an increase in hematocrit (Ht) and/or hemoglobin (Hb) concentration. Among this group of diseases, polycythemia vera arises from a primary acquired clonal abnormality of the red blood cell lineage, leading to a myeloproliferative neoplasm. Its diagnosis has been standardized by the WHO criteria, relying particularly on the presence of a *JAK2* mutation in exons 14 or 12, which is found in nearly 98% of patients [1]. Secondary erythrocytoses are most often acquired and can result from an adaptive response to hypoxia that stimulates erythropoietin (EPO) secretion, from heart and lung diseases, or from some EPO-secreting tumors [2]. Congenital secondary erythrocytosis can result from mutations in the genes encoding (i) high oxygen affinity Hb, (ii) proteins involved in the oxygen-sensing pathway, or, rarely, (iii) bisphosphoglycerate mutase (BPGM), leading to bisphosphoglycerate mutase deficiency. When no classical cause is found, it is usual to refer to the condition as idiopathic erythrocytosis. In recent years, idiopathic erythrocytoses have been better described, thanks in part to the use of genomic sequencing tools [3]. However, despite the use of next generation sequencing (NGS), an etiology is only found in about 25% of idiopathic erythrocytosis cases [4,5].

High oxygen affinity hemoglobin (HOAH) is the main constitutional cause of erythrocytosis [6]. HOAH shows an autosomal dominant transmission, although the absence of a family history does not rule out the diagnosis since there are de novo forms [7]. Pathogenic sequence variations in the genes encoding the alpha or beta globin chains of Hb (*HBA1*, *HBA2* and *HBB*) strengthen the binding of oxygen to the Hb. The oxygen cannot therefore be released to the tissues from the Hb, which brings about hypoxia, increased EPO secretion and thus secondary erythrocytosis. The diagnosis of HOAH is based on the discovery of a specific sequence variation in the alpha or beta globin genes in specialized laboratories, associated with a written and signed consent form.

Nowadays, more than 200 different variants have been identified as responsible for HOAH [8]. Clinical manifestations are highly variable, from asymptomatic forms to major erythrocytosis.

It is important that clinicians recognize HOAH in order to spare the patient multiple unnecessary and expensive complementary examinations and to institute appropriate patient management. Screening tests are of two types based upon the biochemical characteristics of the globin chains or upon the oxygen affinity itself. Therefore, Hb phenotypic studies including electrophoresis or chromatography can be very useful in the characterization of abnormal Hb; nevertheless, a routine electrophoretic approach may fail to detect abnormal Hb, so molecular analysis is essential for establishing a diagnosis [7].

The establishment of the dissociation curve of Hb, which represents the percentage of oxygen-saturated Hb according to the partial pressure of oxygen, makes it possible to perform a direct functional study of Hb. A shift of the curve to the left indicates an increased affinity for Hb. The reference technique is the Hemox Analyzer, which studies Hb oxygen saturation of washed erythrocytes in a buffer. However, due to the very short delivery times for samples and the time-consuming nature of the technique, it is done in very few specialized laboratories. Alternatively, the Hb dissociation can be estimated through venous P50, the oxygen tension at which 50% of Hb is saturated. Indeed, the venous P50, which is normally measured at 27 ± 1 mmHg under physiological conditions, is decreased in HOAH. An estimated measure of the P50 can be obtained inexpensively by most blood gas analyzers.

Previous studies have reported that lowered P50 leads the diagnosis towards either HOAH or a bisphosphoglycerate mutase deficiency [9]. Nevertheless, the validity of venous P50 obtained using blood gas automats has been the subject of controversy due to poor correlation with oximeter measurements. 

While it seems now well established that decreased venous P50 is strongly suggestive of HOAH (or rarely of bisphosphoglycerate mutase deficiency), which is then confirmed by sequencing of globin or *BPGM* genes, it seems difficult to make a statement about its predictive value when within the normal range.

To evaluate the relevance of the venous P50 measured using blood gas automats, we performed genotypic analysis of the *HBB*, *HBA1*, *HBA2* genes on a large series of 75 consecutive patients from various centers in France who had already completed an exhaustive medical check-up in order to compare their results with the venous P50 measures [10,11].

## 2. Materials and Methods

Seventy-five patients from various centers in France with well-characterized idiopathic erythrocytosis were tested. Inclusion criteria implied an exhaustive standard medical check-up. Hence, diagnosis of idiopathic erythrocytosis was confirmed by clinical and biological analyses including measurements of full blood count, blood electrolytes, serum EPO and blood gases, functional respiratory tests, an abdominal ultrasound scan, screening for the *JAK2* mutations on exons 14 and 12 and red cell mass measurement.

None of the patients had *JAK2* mutations or acquired conditions associated with erythrocytosis. They also had no mutations in genes involved (i) in the regulation of the hypoxia pathway (PHD1 (*EGLN2*), PHD2 (*EGLN1*), PHD3 (*EGLN3*), *HIF-1A*, HIF-2A (*EPAS1*), *HIF-3A*, *VHL*, *VHLL*), (ii) in proliferation and differentiation of erythroid progenitors (*EPO*, *EPOR*, *JAK2*, LNK (*SH2B3*), *CBL*), or (iii) in mature cell function (bisphosphoglycerate mutase (*BPGM*)), using a dedicated NGS panel. In 4% of the patients, a *PIEZO1* mutation was noted, leading ultimately to the diagnosis of hereditary xerocytosis with well-compensated hemolysis [12]. As the initial NGS panel did not include *HBB*, *HBA1* and *HBA2* genes encoding the globin chains, DNA samples were tested in a second step for all patients, regardless the P50 value, using another NGS erythrocyte gene panel that included *HBB*, *HBA1* and *HBA2* genes. Each nucleotide variation expected to be probably pathogeneous (class 4) or pathogeneous (class 5 of the ACMG classification ref 2015) was checked using conventional Sanger sequencing. Written consent was obtained from all patients.

## 3. Results

A total of 68 males and seven females (median age 54.6 years, range 9–84 years) were included in our study. Biological data are reported in Table 1. Most of the patients were being treated with phlebotomy for their erythrocytosis. In all the patients, the venous P50 values had been considered as normal in the local centers.

Surprisingly, after the sequencing of the *HBB, HBA1* and *HBA2* genes, HOAH was detected in three patients (Hb Olympia in two patients (Patients #1 and #2), Hb St Nazaire in a third patient (Patient #3)). The two patients with Hb Olympia were from the same family (father and son) and had previously never been detected. As expected, the electrophoresis and chromatography profiles showed no separation between HbA for Hb Olympia. They carry the same nucleotide variation on the *HBB* gene (NM_000518.5:c.61G>A or NP_000509.1:p.(Val21Met)). Due to the discrepancy between the presence of Hb Olympia and the venous P50 value, which was recorded as normal, a careful retrospective examination of the medical files was performed. We finally discovered that the P50 value came not from venous but from arterial blood gas for the two patients. We thus recommended that venous blood gas be analyzed. A decreased venous P50 (21.8 mmHg) was obtained for the father, but the result was unfortunately uninterpretable in the son because there was air in the sample.

For the patient with Hb St Nazaire, the sequencing noted a nucleotide variation on the *HBB* gene (NM_000518.5:c.310T>A or NP_000509.1:p.(Phe104Ile)). The variant hemoglobin is not separated from HbA using standard electrophoresis or chromatography methods, in agreement with the variant description given in the Globin Gene Server (https://globin.bx.psu.edu, accessed on 4 January 2022). Only isoelectric focusing can reveal a slight band broadening of the Hb A band and the highly specific reversed phase HPLC methodology (which is not performed in our lab) is needed to separate the variant from the normal beta chain. He initially was recorded as having normal venous blood P50. After a careful inspection of his medical record, it appeared that venous blood gases had not actually been collected. A venous blood sample was therefore collected, and showed low venous P50, evaluated at 19 mmHg. Biological and clinical data of these three patients are summarized in Table 2.

## 4. Discussion

Idiopathic erythrocytosis is a common condition in clinical hematology and is investigated in the first line with standard biological tests, including *JAK2* mutation (exons 14 and 12), serum EPO, arterial blood gases (to rule out hypoxia or carbon monoxide poisoning) and venous blood gases (to detect HOAH or, rarely, bisphosphoglycerate mutase deficiency). Abdominopelvic ultrasound is also useful to evaluate the presence of splenomegaly, renal abnormalities, uterine leiomyoma or hepatocarcinoma.

Our cohort included 75 patients with idiopathic erythrocytosis who had previously been tested using NGS with a dedicated panel for erythrocytosis. The *HBB*, *HBA1* and *HBA2* genes were not part of our initial NGS panel since in our initial diagnostic strategy, blood gases were performed as part of the screening workup for erythrocytosis. Therefore, in the case of lowered venous P50, simple Sanger sequencing of the *HBB*, *HBA1* and *HBA2* genes was performed. This strategy allowed us to avoid performing complete NGS analysis in all cases since it is a costly and time-consuming test [5].

In the current study, we wanted to check whether our diagnostic approach was relevant and did not overlook cases of HOAH. However, sequencing detected HOAH in three patients: Hb St Nazaire in one patient, and Hb Olympia in two members in the same family (father and son).

Our results underline the fact that the P50 findings were not trustworthy in a small proportion of cases. These discrepancies were not the result of a poor correlation between venous P50 and the presence of high affinity hemoglobin (the two patients with HOAH and evaluable P50 had lowered venous P50 values, i.e., 21.8 and 19 mmHg, respectively); they were associated with the conditions under which the blood gases were taken or with an error on the part of the clinician. The three clinical situations reported here demonstrate that when P50 is tested in arterial blood or when there are technical issues (air bubble in the syringe, for example), it can artificially modify this parameter and therefore falsely eliminate the hypothesis of HOAH. Indeed, due to very short delivery times for samples for the testing of blood gases, it is usually recommended that the tests be performed in the local laboratory. Thus, if there is no guarantee that the pre-analytical and analytical conditions are respected, one could reasonably question whether the P50 is a reliable screening test. On the other hand, except for the decrease in venous P50, it was not possible to distinguish patients with HOAH from other causes of idiopathic erythrocytosis, only on the parameters of routine biology.

In a very interesting study published by Oliveira et al. from a large series of patients with HOAH Hb (*n* = 762), a P50 < 24 mmHg was found in almost 99% of patients. In other words, the probability of having HOAH with a P50 ≥ 24 is about 1%, which makes this parameter a good screening test [4].

Our results, though based on a smaller series of patients, are in accordance with those from Oliveira et al., confirming that the diagnosis of HOAH is unlikely if venous P50 is normal and the analytical conditions are good. It should be noted that the venous oxygen saturation should be between 30% and 55 % to validate that P50 was tested in venous blood and thus confirm its use as a relevant biological parameter [13]. This range could be used at least as a verification parameter or as a warning that the analytical conditions were not adequate.

## 5. Conclusions

Finally, our results suggest that, even in a cohort of clinically and biologically well-characterized patients with idiopathic erythrocytosis, the hypothesis of high affinity hemoglobin cannot be ruled out on the sole value of the P50 due to potential pre-analytical and analytical hazards (use of arterial blood gas, presence of air bubbles in the syringe, etc.). At a time when molecular genetic tests are being generalized, our study strongly suggests that the *HBB*, *HBA1* and *HBA2* genes must be included in the panel of genes for the screening of familial erythrocytosis, in order to increase the sensitivity of the diagnosis even when blood gas tests appear to be normal.

## Figures and Tables

**Table 1:** Clinical and biological data of the patients.

	Number of Patients	Sex	Age	Hb * (g/dL)	Ht * (%)	RBC (*1012/L)	EPO (UI/L)	Red Cell Mass * (%)	p50 (mmHg)	White Blood Cells (G/L)	Platelets (G/L)
**Median value**	75.0	90.6% M (*n* = 68); 9.3% F (*n* = 7)	58.0	18.1	53	6.1	8.3	135	26.8	6.9	210.0
**Lower value**			9.0	14.1	43.8	5.0	1.5	110	20.6	3.9	93.0
**Higher value**			84.0	28.0	70.0	8.6	40.0	143	33.0	10.2	372.0
**Reference values**				12–16 F 14–18 M	37–47 F 42–54 M	4.2–5.2 F 4.5–5.7 M	8–30 F 5.6–28.9 M	80–125	25–27	4–10	150–450

Hb = Hemoglobin; Ht = Hematocrit; RBC = Red Blood Cell; M = Male; F = Female; EPO = Erythropoietin. * Some of the patients had undergone bloodletting before the analyses, that may have affected the results of some parameters such as EPO, MCV. As far as possible, most of the analyses were performed before the bloodlettings.

**Table 2:** Clinical and biological data of the 3 patients.

Patient #	Age	Hb (g/dL)	Ht (%)	Red Blood Cells (T/L)	EPO (UI/L)	Red Cell Mass (%)	Venous P50 (mmHg)	White Blood Cells (G/L)	Platelets (G/L)	Familial History	HOAH Variant
1	70	17.7	53.3	6.0	13.1	136	21.8	8.5	237.0	Yes (Brother and Son)	c.61G > A/ p.(Val21Met)
2	42	14.1 (after 3 phlebotomies)	43.8	U	7.0	128		6.8	372.0	Yes (Father and Uncle)	c.61G > A/ p.(Val21Met)
3	39	19.0	56.6	6.1	U	144	19.0	5.4	187.0	Yes (Mother, Aunt)	c.310T > A/ p.(Phe104Ile)

Hb = Hemoglobin; Ht = Hematocrit; EPO = Erythropoietin; U = Unknown.

## Data Availability

The data presented in this study are available on request from the corresponding author. The data are not publicly available due to ethical restrictions.
